# Gene mutation in diabetic patients with lung adenocarcinoma: a real-world retrospective cohort study

**DOI:** 10.3389/fmed.2025.1460956

**Published:** 2025-01-24

**Authors:** Lei Yang, Yang Hong, TingTing Zeng, HongMei Yue, DePeng Jiang

**Affiliations:** ^1^Department of Respiratory Medicine, The Second Affiliated Hospital of Chongqing Medical University, Chongqing, China; ^2^Department of Endocrinology, The Second Affiliated Hospital of Chongqing Medical University, Chongqing, China; ^3^Department of Respiratory Medicine, The First Affiliated Hospital of Lanzhou University, Lanzhou, China

**Keywords:** lung adenocarcinoma, gene mutations, diabetes, smoking, drinking

## Abstract

**Purpose:**

The incidence of lung cancer is closely associated with diabetes; however, it remains unclear whether diabetes influences the genetic mutations present in lung cancer. Therefore, we will compare the genetic mutations in patients with lung adenocarcinoma (ADC) who have diabetes against those who do not.

**Methods:**

We included 279 patients diagnosed with lung adenocarcinoma (143 with diabetes and 136 without diabetes) at the Second Affiliated Hospital of Chongqing Medical University between 2016 and 2023, and analyzed the clinical characteristics and genetic mutation profiles of all participants.

**Results:**

In comparison to ADC patients without diabetes, those with diabetes exhibited a lower overall gene mutation rate (49.7% vs. 65.4%, *P* = 0.008). Female ADC patients demonstrated a higher total gene mutation rate and EGFR gene mutation rate than their male counterparts (49.3% vs. 66.9%, *P* = 0.003; 27.6% vs. 58.3%, *P* < 0.001, respectively), although their TP53 gene mutation rate was lower (8.6% vs. 2.4%, *P* = 0.027). ADC patients without a smoking history had a higher gene mutation rate and EGFR gene mutation rate than those with a smoking history (62.6% vs. 47.4%, *P* = 0.014; 51.6% vs. 22.7%, *P* < 0.001, respectively), but a lower KRAS gene mutation rate (4.4% vs. 14.4%, *P* = 0.003). Conversely, ADC patients with a drinking history had a lower EGFR gene mutation rate than those without (48% vs. 62.6%, *P* = 0.018; 31.0% vs. 47.5%, *P* = 0.007), yet a higher KRAS gene mutation rate (14.0% vs. 4.5%, *P* = 0.005). Univariate and multivariate linear regression analyses revealed that being female, having no smoking history, and being in phase II or IV of tumor stage were associated with gene mutation. Subgroup analysis indicated that the rate of gene mutation in male smoking lung adenocarcinoma patients with diabetes was significantly lower than in those without diabetes.

**Conclusion:**

This retrospective study of real-world data suggests that patients with lung adenocarcinoma and diabetes may have a reduced likelihood of developing genetic mutations, particularly among male smokers. Furthermore, gender, smoking history, and tumor stage may be correlated with the presence of gene mutations.

## Introduction

Lung cancer stands as the second most prevalent form of malignant cancer, responsible for 11.4% of all new cancer diagnoses. It remains the primary cause of cancer-related mortality, with an estimated 1.8 million fatalities annually (1). Lung adenocarcinoma (ADC) has emerged as the predominant cell type among lung cancer cases globally (2). Interestingly, East Asians who have never smoked are more frequently diagnosed with adenocarcinoma, a subtype characterized by specific oncogenic drivers. The discovery of activating epidermal growth factor receptor (EGFR) mutations, which respond to EGFR tyrosine kinase inhibitors (TKIs), was initially made in Asian women and non-smokers with lung adenocarcinoma (3–5). As high-throughput sequencing technology has progressed, the molecular landscape of lung cancer has unveiled a spectrum of carcinogenic factors. Consequently, genetic testing is now advised for all patients newly diagnosed with non-small cell lung cancer (NSCLC), encompassing mutations in EGFR, anaplastic lymphoma kinase (ALK), ROS proto-oncogene 1 (ROS1), Rearranged during transfection (RET), B-Raf proto-oncogene, serine/threonine kinase (BRAF) V600E, and MET exon 14 skipping mutations. Additionally, the testing should include the evaluation of gene amplifications or overexpression's, such as those in MET, human epidermal growth factor receptor 2 (HER2), Kirsten rat sarcoma viral oncogene homolog (KRAS), and NeuroTrophin Receptor Kinase (NTRK) (6–8).

Diabetes mellitus (DM), the most prevalent metabolic disorder, is characterized by chronically elevated blood glucose levels. This condition manifests in two distinct pathological forms: type 1 diabetes (T1DM) and type 2 diabetes (T2DM). Research has established a bidirectional relationship between diabetes and cancer, with type 2 diabetes in particular being associated with a heightened risk of developing cancer (9). Among the various types of cancer, lung cancer is the most frequent in individuals with type 2 diabetes, with adenocarcinoma being the predominant form (10). Irrespective of the diabetes type, elevated blood glucose can precipitate a range of pulmonary complications, including asthma, chronic obstructive pulmonary disease (COPD), pneumonia, fibrosis, and lung cancer (LC) (11). Multiple potential mechanisms, such as hyperglycemia, hyperinsulinemia, glycation, inflammation, and hypoxia, have been proposed as possible connections between DM and LC (12).

DM is linked to numerous genetic mutations in disease-causing genes. Research has indicated that diabetes appears to elevate the risk of BRAF mutations in patients with colorectal cancer, a condition typically associated with a poor prognosis (13). Furthermore, patients with bone marrow syndrome who also have diabetes exhibit a higher mutation rate of the 10-11 translocation 2 (TET2) and splicing factor 3b subunit 1 gene (SF3B1), which correlates with a more severe prognosis (14). Another study has confirmed that diabetes can dynamically influence tuberculosis (TB) drug resistance genes (15).

However, the impact of diabetes on gene mutation in patients with lung adenocarcinoma remains uncertain. The primary objective of this study was to investigate whether there exist any differences in tumor gene mutations between lung adenocarcinoma patients with diabetes and those without diabetes. Secondary objectives encompass identifying the ways in which various diabetes medications interact with genetic mutations in patients diagnosed with lung adenocarcinoma. To delve into these distinctions, we gathered and analyzed data from 279 patients who had been pathologically diagnosed with lung adenocarcinoma and had undergone genetic testing.

## Materials and methods

### Subject investigated

**Figure 1:**
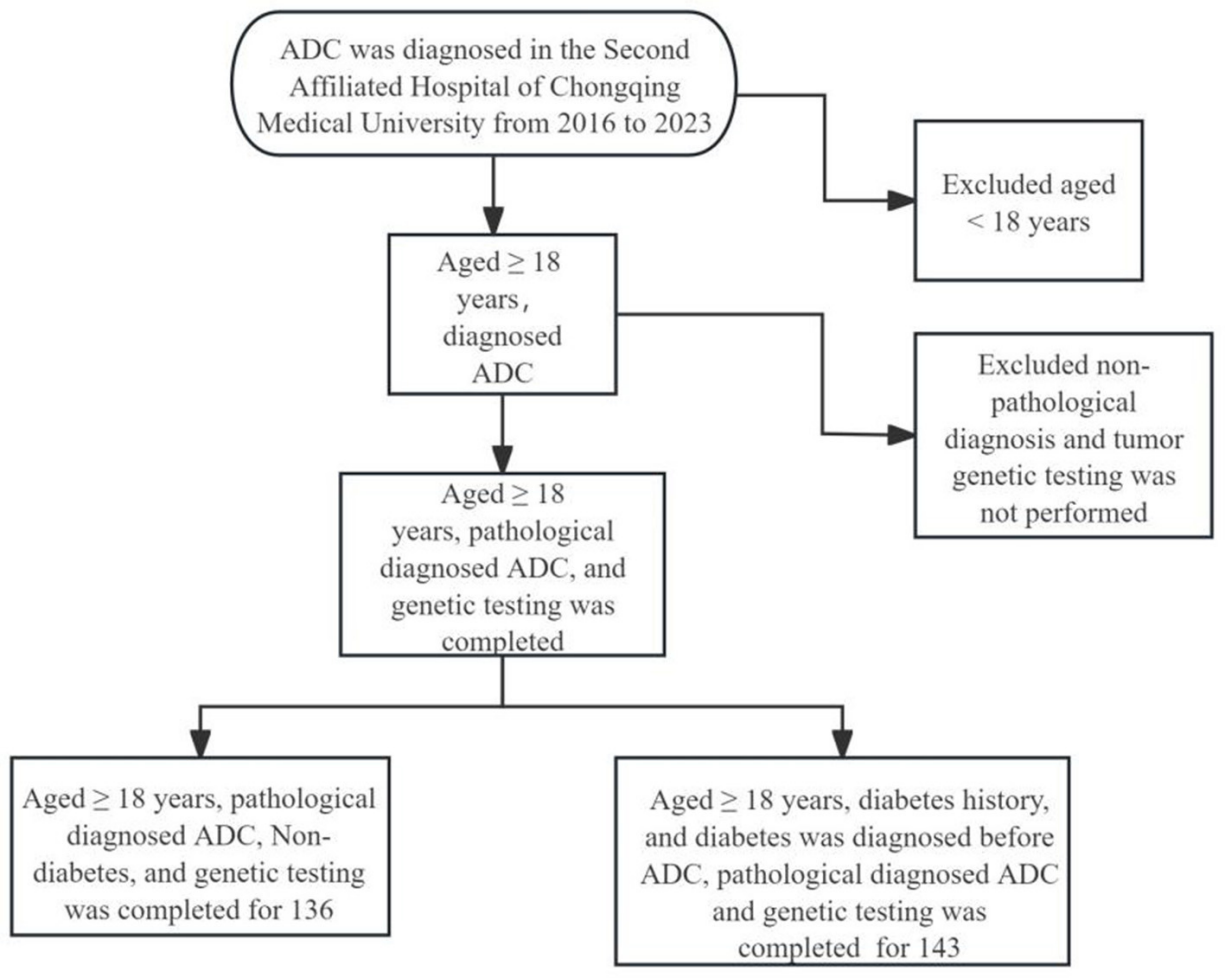
Flowchart of participant selection. ADC, lung adenocarcinoma.

A retrospective analysis was conducted on patients with lung adenocarcinoma diagnosed by the Department of Respiratory Medicine, Oncology, and Thoracic Surgery between 2016 and 2023. The duration of diabetes ranged from 1 to 20 years, and all patients were diagnosed of type 2 diabetes and exhibited mild symptoms. The inclusion criteria were as follows: (1) Age of 18 years or older; (2) Pathological diagnosis of lung adenocarcinoma; (3) Completion of tumor gene testing; (4) A history of diabetes, with or without; (5) Diagnosis of diabetes preceding that of lung adenocarcinoma. The exclusion criteria were: (1) Age younger than 18 years; (2) Incomplete tumor genetic testing, as shown in Figure 1. All participants provided signed informed consent forms.

#### Mutation gene

All genes were subjected to testing and analysis using Next-generation sequencing (NGS), a process conducted in China. The study encompassed commonly mutated genes in lung adenocarcinoma, including EGFR, KRAS, ALK, MET, BRAF, HER2, Tumor Protein 53 (TP53), phosphoinositide 3-kinase (PIK3), and avian Erythroblastosis oncogene B 2 (ERBB2).

### Statistical method

The analysis of ordinal variables utilized the median as the primary measure of central tendency. For the examination of categorical variables, the chi-square test or Fisher's exact test was chosen as the preferred method. To assess continuous variables, both the Mann–Whitney *U*-test and the independent samples *T*-test were employed. In order to identify with a high degree of certainty the factors influencing gene mutations, both univariate and multivariate ordinal linear regression analyses were conducted. The statistical computations were performed using SPSS 26 software, which is an offering of IBM Corp. located in Armonk, NY, USA. Additionally, GraphPad Prism (version 9.5.1) was utilized for plotting purposes. A *P* value of less than 0.05 was deemed indicative of a statistically significant difference.

## Results

### General situation and basic characteristics

This study compiled a total of 279 cases, comprising 143 ADC patients with diabetes and 136 ADC patients without diabetes. As presented in Table 1, no significant disparities were observed in gender, alcohol consumption history, hemoglobin levels, leukocyte count, creatinine levels, alanine aminotransferase (ALT), aspartate aminotransferase (AST), carcinoembryonic antigen (CEA), cytokeratin 19 fragments (CYFRA211), and neuron-specific enolase (NSE) between the ADC patients with diabetes and the control group. However, the ADC patients with diabetes exhibited a higher median body mass index (BMI) (23.19 vs. 21.77, *P* < 0.001) and lower platelet counts (210 vs. 236, *P* = 0.029). Additionally, notable differences were identified in the clinical stage and nodule characteristics between the two groups.

**Table 1:** General characteristics of all the participants.

**Various**	**ADC patients with diabetes (*n* = 143)**	**ADC patients without diabetes (*n* = 136)**	***P*-value**	*P*-value^*^
Age (years) mean (SD) median (Min-Max)	67.57 (8.89)	65.66 (8.85)	0.074	0.076
	68.00 (40.00–86.00)	66.00 (45.00–91.00)		
BMI (Kg/m^2^) mean (SD) median (Min-Max)	23.36 (3.01)	22.00 (3.07)	< 0.001	< 0.001
	23.19 (15.82–30.30)	21.77 (13.78–31.11)		
HB (g/L) mean (SD) median (Min-Max)	121.88 (16.71)	123.70 (18.29)	0.387	0.210
	123.00 (87.00–169.00)	126.00 (61.00–172.00)		
WBC (10∧9/L) mean (SD) median (Min-Max)	7.02 (2.22)	6.82 (1.88)	0.423	0.807
	6.60 (2.89–15.81)	6.69 (3.49–13.42)		
PLT (10∧9/L) mean (SD) median (Min-Max)	220.69 (85.38)	243.85 (91.02)	0.029	0.025
	210.00 (25.00–511.00)	235.50 (11.00–485.00)		
AST (IU/L) mean (SD) median (Min-Max)	21.23 (14.19)	19.60 (12.01)	0.301	0.347
	17.00 (4.00–115.00)	17.00 (6.00–81.00)		
ALT (IU/L) mean (SD) median (Min-Max)	24.09 (25.99)	21.92 (8.76)	0.355	0.416
	19.00 (4.00–269.00)	20.50 (8.00–60.00)		
CR(μmol/L) mean (SD) median (Min-Max)	70.92 (46.04)	65.68 (17.23)	0.213	0.957
	63.10 (5.90–418.80)	63.05 (30.50–130.60)		
Carcinoembryo nic antigen mean (SD) median (Min-Max)	89.86 (214.11)	66.18 (180.92)	0.320	0.397
	7.92 (0.41–1000.00)	7.47 (0.23–1000.00)		
Cytokeratin 19 fragments mean (SD) median (Min-Max)	5.65 (7.25)	7.77 (14.51)	0.121	0.745
	3.21 (0.15–58.91)	3.08 (0.76–100.00)		
Neuron-specific enolase mean (SD) median (Min-Max)	15.18 (6.63)	17.19 (11.78)	0.078	0.492
	13.91 (7.29–65.81)	13.98 (1.90–84.56)		
**Sex**				
Male	82 (57.34%)	70 (51.47%)	0.325	
Female	61 (42.66%)	66 (48.53%)		
**Smoking history**				
Yes	43 (30.07%)	54 (39.71%)	0.091	
No	100 (69.93%)	82 (60.29%)		
**Drinking history**				
Yes	59 (41.26%)	41 (30.15%)	0.053	
No	84 (58.74%)	95 (69.85%)		
**Tumor location**				
Up left	35 (24.48%)	31 (22.79%)	0.348	
Low left	28 (19.58%)	24 (17.65%)		
Upper right	44 (30.77%)	40 (29.41%)		
Center right	14 (9.79%)	7 (5.15%)		
Low right	18 (12.59%)	29 (21.32%)		
Hilus of the lung	4 (2.80%)	5 (3.68%)		
**Nodule property**				
Solid nodules	121 (84.62%)	130 (95.59%)	0.002	< 0.001
Ground glass nodules	17 (11.89%)	2 (1.47%)		
Mixed nodules	5 (3.50%)	4 (2.94%)		
**Clinical stages**				
I	36 (25.17%)	9 (6.62%)	< 0.001	
II	7 (4.90%)	4 (2.94%)		
III	10 (6.99%)	13 (9.56%)		
IV	90 (62.94%)	110 (80.88%)		

^*^Presented as Median (Range). For categorical variables, Fisher's Exact Test and the Chi-Square Test of Independence were employed, whereas for continuous variables, the Mann-Whitney *U*-Test and the *T*-Test were utilized.

### Gene mutation characteristics for all participators

ADC patients with DM exhibited a lower total gene mutation rate compared to those without DM (49.7% vs. 65.4%, *P* = 0.008), as indicated in Table 2. Among female ADC patients, the gene mutation rate and EGFR gene mutation rate were higher than in male patients (49.3% vs. 66.9%, *P* = 0.003; 27.6% vs. 58.3%, *P* < 0.001, respectively). However, the TP53 gene mutation rate was lower in females (8.6% vs. 2.4%, *P* = 0.027), as detailed in Table 3. ADC patients without a smoking history had a higher gene mutation rate and EGFR gene mutation rate than those with a smoking history (62.6% vs. 47.4%, *P* = 0.014; 51.6% vs. 22.7%, *P* < 0.001, respectively), but a lower KRAS gene mutation rate (4.4% vs. 14.4%, *P* = 0.003), as presented in Table 4. Conversely, ADC patients with a drinking history had a lower gene mutation rate and EGFR gene mutation rate than those without a drinking history (48% vs. 62.6%, *P* = 0.018; 31.0% vs. 47.5%, *P* = 0.007), yet a higher KRAS gene mutation rate (14.0% vs. 4.5%, *P* = 0.005), as outlined in Table 5.

**Table 2:** Comparison of gene mutations in ADC patients with or without diabetes.

	**ADC patients with diabetes (*n* = 143)**	**ADC patients without diabetes (*n* = 136)**	***P*-value**
Total gene mutation rate (%)	49.7% (71/143)	65.4% (89/136)	0.008
EGFR (%)	37.8% (54/143)	45.6% (62/136)	0.185
ALK (%)	1.4% (2/143)	4.4% (6/136)	0.164
KRAS (%)	7.7% (11/143)	8.1% (11/136)	0.902
MET (%)	2.1% (3/143)	/	1
BRAF (%)	0.7% (1/143)	1.5% (2/136)	1
HER2 (%)	0.7% (1/143)	1.5% (2/136)	1
TP53 (%)	4.2% (6/143)	7.35% (10/136)	0.257
PTK3 (%)	0.7% (1/143)	1.5% (2/136)	1
ERBB2 (%)	1.4% (2/143)	1.5% (2/136)	1

Fisher's exact test and the Chi-Square statistic test were utilized.

**Table 3:** Comparison of gene mutation in ADC patients of different gender.

	**ADC patients (male)**	**ADC patients (female)**	***P***
Total gene mutation rate (%)	49.3% (75/152)	66.9% (85/127)	0.003
EGFR (%)	27.6% (42/152)	58.3% (74/127)	< 0.001
ALK (%)	2.6% (4/152)	3.1% (4/127)	1
KRAS (%)	9.9% (15/152)	5.5% (7/127)	0.179
MET (%)	1.3% (2/152)	0.8% (1/127)	1
BRAF (%)	1.3% (2/152)	0.8% (1/127)	1
HER2 (%)	2.0% (3/152)	/	1
TP53 (%)	8.6% (13/152)	2.4% (3/127)	0.027
PIK3 (%)	1.3% (2/152)	0.8% (1/127)	1
ERBB2 (%)	1.3% (2/152)	1.6% (2/127)	1

Fisher's exact test and the Chi-Square statistic test were utilized.

**Table 4:** Comparison of gene mutation in ADC patients with or without smoking history.

	**ADC patients without smoking history**	**ADC patients with smoking history**	***P*-value**
Total gene mutation rate (%)	62.6% (114/182)	47.4% (46/97)	0.014
EGFR (%)	51.6% (94/182)	22.7% (22/97)	< 0.001
ALK (%)	3.3% (6/182)	2.1% (2/97)	0.718
KRAS (%)	4.4% (8/182)	14.4% (14/97)	0.003
MET (%)	0.5% (1/182)	2.1% (2/97)	1
BRAF (%)	1.1% (2/182)	1.0% (1/97)	1
HER2 (%)	/	3.1% (3/97)	1
TP53 (%)	6.0% (11/182)	5.6% (5/97)	0.761
PTK3 (%)	1.6% (3/182)	/	1
ERBB2 (%)	1.6% (3/182)	1.0% (1/97)	1

Fisher's exact test and the Chi-Square statistic test were utilized.

**Table 5:** Comparison of gene mutation in ADC patients with or without drinking history.

	**ADC patients with drinking history**	**ADC patients without drinking history**	***P*-value**
Total gene mutation rate (%)	48.0% (48/100)	62.6% (112/179)	0.018
EGFR (%)	31.0% (31/100)	47.5% (85/179)	< 0.001
ALK (%)	2.0% (2/100)	3.4% (6/179)	0.716
KRAS (%)	14.0% (14/100)	4.5% (8/179)	0.005
MET (%)	1.0% (1/100)	1.1% (2/179)	1
BRAF (%)	1.0% (1/100)	1.1% (2/179)	1
HER2 (%)	2.0% (2/100)	0.6% (1/179)	1
TP53 (%)	7.0% (7/100)	5.0% (9/179)	0.497
PTK3 (%)	/	1.7% (3/179)	1
ERBB2 (%)	1.0% (1/100)	1.7% (3/179)	1

Fisher's exact test and the Chi-Square statistic test were utilized.

### Univariate and multivariate analysis

Upon conducting univariate and multivariate regression analyses, we determined that gender, smoking history, and clinical stage were independent risk factors for genetic mutations in ADC patients with diabetes, with a significance level of *P* < 0.05 as shown in Table 6.

**Table 6:** Univariate and multivariate analysis of ADC patients with diabetes.

	**Univariate OR (95% CI)**	***P* value**	**Multivariate OR (95% CI)**	***P* value**
Age (years)	1.017 (0.980, 1.056)	0.368	1.038 (0.982, 1.098)	0.184
Gender (female)	1.933 (0.987, 3.787)	0.055	4.112 (0.995, 16.988)	0.051
Smoking history (No)	2.746 (1.296, 5.817)	0.008	9.290 (2.104, 41.028)	0.003
Drinking history (No)	1.646 (0.841, 3.223)	0.146	0.375 (0.077, 1.828)	0.225
Body mass index (Kg/m^2^)	0.976 (0.875, 1.089)	0.669		
Hemoglobin (g/L)	0.979 (0.959, 0.999)	0.04	0.983 (0.954, 1.013)	0.273
White blood cell (10∧9/L)	1.030 (0.888, 1.194)	0.698		
Platelet (10∧9/L)	1.001 (0.996, 1.003)	0.826		
Aspartate transaminase (IU/L)	0.999 (0.976, 1.022)	0.93		
Alanine aminotransferase (IU/L)	1.003 (0.990, 1.017)	0.619		
Creatinine (μmol/L)	1.005 (0.996, 1.014)	0.277		
Carcinoembryonic antigen	1.001 (1.000, 1.003)	0.16	0.999 (0.997, 1.002)	0.492
Cytokeratin 19 fragments	1.071 (1.002, 1.144)	0.042	1.052 (0.958, 1.156)	0.285
Neuron-specific enolase	1.038 (0.979, 1.010)	0.208	1.007 (0.913, 1.109)	0.894
Glycated hemoglobin	0.958 (0.753, 1.219)	0.729	0.825 (0.549, 1.240)	0.355
**Tumor location**				
Low left	0.891 (0.327, 2.424)	0.821	1.079 (0.254, 4.585)	0.917
Upper right	1.301 (0.534, 3.167)	0.563	1.956 (0.496, 7.718)	0.338
Center right	1.583 (0.454, 5.527)	0.471	1.092 (0.165, 7.242)	0.927
Low right	1.484 (0.473, 4.656)	0.498	1.290 (0.249, 6.677)	0.762
Hilus of the lung	1.188 (0.150, 9.408)	0.871	0.861 (0.0197, 37.626)	0.938
**Nodule property**				
Ground glass nodules	0.857 (0.069, 10.667)	0.905	3.529 (0.113, 110.208)	473
Solid nodules	4.963 (0.539, 45.715)	0.157	9.688 (0.420, 223.419)	0.156
**Diabetes treatment**				
Insulin	1.942 (0.734, 5.141)	0.181	1.193 (0.235, 6.0444)	0.832
Oral hypoglycemic drugs + Insulin	1.195 (0.281, 5.078)	0.809	1.744 (0.148, 20.546)	0.658
Diet control + exercise	1.412 (0.572, 3.487)	0.454	1.007 (0.229, 4.422)	0.993
Disease interval time (years)	1.087 (0.792, 1.490)	0.606	1.215 (0.695, 2.122)	0.494
**Clinical stage**				
II	6.667 (1.176, 37.781)	0.032	17.798 (1.975, 160.383)	0.01
III	0.556 (0.059, 5.241)	0.608	0.989 (0.074, 13.187)	0.993
IV	9.833 (3.689, 26.215)	< 0.001	16.939 (4.368, 65.692)	< 0.001

OR, odds ratio; CI, confidence interval.

### Subgroup analysis

**Figure 2:**
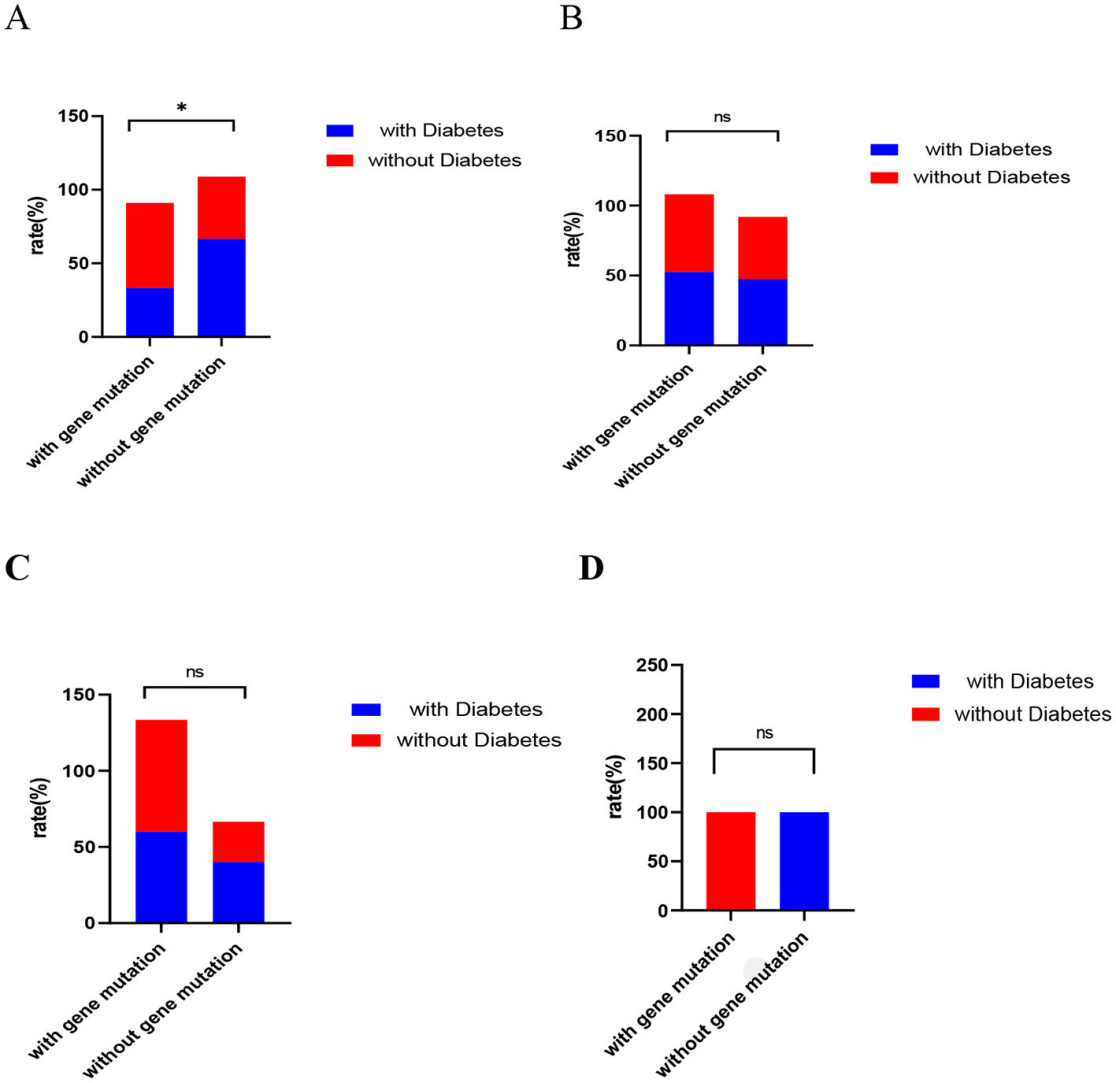
Comparison of gene mutations in ADC patients with or without diabetes in subgroup analysis. The gene mutation rate among patients with lung adenocarcinoma was found to be intermediate between those with and without diabetes [male smokers, **(A)**; male non-smokers, **(B)**; female non-smokers, **(C)**; female smokers, **(D)**]. Statistical analyses were conducted using Fisher's exact test and the Chi-Square test, with “ns” denoting *P* > 0.05, and *P* < 0.05 indicated by an asterisk (“*”).

In further subgroup analysis, for the patient population with ADC among male smokers, we observed a reduced rate of gene mutation patients with diabetes compared to those without diabetes (*P* = 0.019) (Figure 2A) shows, which is no difference in other subgroups (Figures 2B–D).

## Discussion

In this retrospective study, we examined the prevalence of common mutated genes in lung adenocarcinoma. We assessed variations in gene mutation rates among patients with lung adenocarcinoma, stratified by the presence or absence of diabetes, smoking history, alcohol consumption, and gender. Our findings indicate that patients with diabetes exhibited a lower rate of gene mutations. Furthermore, these mutations were associated with sex, smoking history, and the stage of the tumor.

Cancer is progressively emerging as the leading cause of mortality globally, with annual projections indicating a surge in both new cases and fatalities (16). An ever-growing body of evidence underscores a direct link between diabetes and cancer, particularly in the context of several of the most prevalent malignant tumors. Prior research has revealed lung cancer as the most frequent malignant tumor that is complicated by diabetes (17). The activation of the oncogene KRAS2, the deactivation of the tumor suppressor gene (Recombinant Cyclin Dependent Kinase Inhibitor 2A, CDKN2A), the silencing of the tumor suppressor TP53, and the mutation of the pancreatic cancer-related gene 4 (DPC4), which holds a pivotal role in pancreatic carcinogenesis, are all intimately associated with a poor prognosis in patients diagnosed with pancreatic cancer. Strikingly, an astonishing 80% of these patients concurrently grapple with diabetes, a figure that underscores the intricate interplay between these genetic mutations and metabolic disturbances (18). Concurrently, patients with colorectal cancer harboring BRAF mutations typically exhibit a poor prognosis, and diabetes further elevates the risk of such mutations (13). In individuals with bone marrow syndrome, mutations in TET2 and SF3B1 genes are associated with a more severe prognosis, and the presence of diabetes also amplifies the frequency of these genetic alterations (14). In our study, the overall gene mutation rate among ADC patients with diabetes was found to be lower (49.7% vs. 65.4%, *P* = 0.008). Moreover, male smokers diagnosed with lung adenocarcinoma and diabetes presented a reduced rate of gene mutations compared to their non-diabetic counterparts (33.3% vs. 57.7%, *P* = 0.019). Consequently, we deduce that the decreased gene mutation rate in patients with diabetes and lung adenocarcinoma may be influenced by a multitude of factors, encompassing genetic predispositions, environmental influences, pharmacological interventions, and immune system status.

However, no significant difference was observed in the mutation rate of individual genes, which contradicts findings from previous studies. This discrepancy may be attributed to the limited sample size of our research or to factors such as smoking habits and gender. The primary risk factors for lung cancer include smoking, yet it is crucial to consider other factors due to the rising incidence of lung cancer in non-smokers (LCINS) (19, 20). Numerous studies (21–23) have indicated a higher prevalence of EGFR mutations in lung cancer patients who have never smoked. Conversely, KRAS mutations are notably more common in patients with a history of tobacco use, and these mutations are often associated with resistance to EGFR-tyrosine kinase inhibitors (24). These results indicate that the two carcinogenic mutations are mutually exclusive. Women have a higher risk of developing lung cancer, especially in lung adenocarcinoma (25). Our research aligns demonstrating that women have significantly greater odds of exhibiting an EGFR mutation in lung tumor tissue compared to men. Previous studies by Chapman and Dang (26, 27) have also corroborated this result, suggesting that women are at a higher risk for lung cancer mutations. According to a study in the Cell that the East Asian EGFR mutation rate was 85%, with a female majority, meanwhile, further analysis of the genetic variants by different sex and smoking status revealed that besides the expected mutual exclusivity between EGFR and KRAS mutations, the RNA-binding motif protein 10(RBM 10) mutations together with the TP53, KRAS, xin actin-binding repeat containing 2(XIRP 2), and zinc finger protein 804B(ZNF804B) mutations were also mutually exclusive. The presence of these mutation exclusivity with high mutation frequency may indicate new synthetic lethality between them or the presence of unique clonal evolution (28–30). Despite the significantly higher prevalence of EGFR mutations in female non-smokers and in patients with women-predominant non-small cell lung cancer (NSCLC), it has been suggested that restricting screenings to only never-smoking women would overlook 57% of all EGFR mutations. The primary reason for this is that a significant percentage of EGFR mutations are found in male patients and smokers, suggesting a broader distribution than previously thought (31). TP53 is linked to the prognosis of lung tumors, and its mutations are indicative of a poor prognosis. Previous studies (32) have indicated that TP53 mutations were present in 125 cases and were significantly correlated with male gender. Our study yielded consistent results, suggesting that the prognosis for male lung adenocarcinoma patients may be less favorable. Furthermore, our findings indicate that lung adenocarcinoma patients with a history of alcohol consumption have a lower rate of EGFR mutations and a higher rate of KRAS mutations. However, factor analysis did not reveal a correlation between alcohol consumption history and gene mutations. Interestingly, our results align with those of studies on smoking history, leading us to suspect that a large proportion of the Chinese population has a history of both smoking and drinking.

The relationship between gene mutations and tumor stage is complex. Previous research has confirmed that EGFR mutations are found in all stages of NSCLC (33). Mutations within exons E18 to E21 were frequently observed in patients diagnosed with lung cancer at stages IA, IB, IIA, and IIB, respectively. Notably, the incidence of KRAS gene mutations in exon E2 was elevated in both whole blood and tissue specimens compared to other exon mutations. Additionally, a significant increase in the frequency of KRAS gene mutations was noted in patients with stage IIB lung cancer within exon E2, and in those with stage IA lung cancer within exon E3 (34). This suggests a potential correlation between gene mutation and the clinical stage of lung cancer. In contrast to previous studies, our univariate and multivariate analyses revealed that clinical stage was associated with gene mutation in lung adenocarcinoma patients with diabetes, with stages II and VI showing a higher likelihood of gene mutation. The analysis may be attributed to population differences or a small sample size. Sex, smoking history, and tumor stage influence genetic mutations and are influenced by genetic and environmental factors.

Sex, smoking, tumor stage, and gene mutation (GSTGM) significantly influence the treatment outcomes and prognosis of lung adenocarcinoma patients. Female patients generally respond better to treatment and have longer survival times, likely due to healthier lifestyle choices (35). Furthermore, smokers are more likely to develop resistant genetic mutations (36) that limit treatment options and increase complications. Meanwhile, early-stage tumors are typically surgically removed, while intermediate and advanced stages require a comprehensive approach. Targeted therapies for specific genetic mutations have proven effective in improving survival rates (37, 38). Personalized treatment plans should involve a thorough assessment of the patient's overall health alongside multidisciplinary expertise for optimal management. Promoting healthy lifestyles and addressing psychological wellbeing is essential for enhancing treatment effectiveness and quality of life.

Diabetes and lung adenocarcinoma are linked through gene mutations and complex interactions. High blood sugar levels can damage enzymes and DeoxyriboNucleic Acid (DNA), potentially causing tumors, and provide energy for cancer cell growth (39, 40). Insulin resistance disrupts metabolism, alters cytokine levels, and can stimulate tumor growth while inhibiting cell death (41). Elevated insulin levels in diabetes can also enhance the effects of Insulin-like Growth Factor (IGF) and Vascular Endothelial Growth Factor (VEGF), promoting tumor cell proliferation (42). In short, diabetes may affect cell cycle, apoptosis, and DNA repair, causing mutations in related genes, contributing to the tumor's development.

The current investigation was subject to several limitations. To begin with, it was a single-center retrospective study conducted in Chongqing, China, which featured a small sample size. Secondly, the study's inclusion of mutated genes was incomplete, and it did not collect data on specific medication regimens for diabetes. Thirdly, the prognosis of all participants was not evaluated or analyzed in our research. Fourthly, there may have been a few errors in the data collection process. Fifthly, there may be other risk factors influencing genetic mutation, such as chronic obstructive pulmonary disease (COPD), tuberculosis, and Interstitial Lung Disease (ILD). Despite these limitations, the study also boasts several benefits: firstly, it is the first study to explore the relationship between diabetes and lung tumor gene mutations. Secondly, it reaffirmed the relationship between smoking history, gender, and lung cancer gene mutations.

In conclusion, patients with lung adenocarcinoma who also have diabetes may exhibit a reduced rate of gene mutation, particularly among male smokers. Gender, smoking history, and clinical stage are associated with gene mutation rates. However, the precise mechanisms of action remain to be fully understood. To achieve a deeper comprehension of this matter, additional basic research is required to elucidate the interactions between diabetes and lung adenocarcinoma and the fundamental reasons behind alterations in gene mutation rates.

## Data Availability

The data analyzed in this study is subject to the following licenses/restrictions: The raw data supporting the conclusions of this article will be made available by the authors, without undue reservation. Requests to access these datasets should be directed to 1946966229@qq.com.

## References

[1] Sung H Ferlay J Siegel RL Laversanne M Soerjomataram I Jemal A. Global Cancer Statistics 2020: GLOBOCAN estimates of incidence and mortality worldwide for 36 cancers in 185 countries. CA Cancer J Clin. (2021) 71:209–49. 10.3322/caac.21660 33538338

[2] Nakamura H Saji H. Worldwide trend of increasing primary adenocarcinoma of the lung. Surg Today. (2014) 44:1004–12. 10.1007/s00595-013-0636-z 23754705

[3] Sun Y Ren Y Fang Z Li C Fang R Gao B. Lung adenocarcinoma from East Asian never-smokers is a disease largely defined by targetable oncogenic mutant kinases. J Clin Oncol. (2010) 30:4616–20. 10.1200/JCO.2010.29.6038 20855837 PMC2974342

[4] Couraud S Zalcman G Milleron B Morin F Souquet PJ. Lung cancer in never smokers–a review. Eur J Cancer. (2012) 48:1299–311. 10.1016/j.ejca.2012.03.007 22464348

[5] Subramanian J Govindan R. Lung cancer in never smokers: a review. J Clin Oncol. (2007) 25:561–70. 10.1200/JCO.2006.06.8015 17290066

[6] Kalemkerian GP Narula N Kennedy EB Biermann WA Donington J Leighl NB. Molecular testing guideline for the selection of patients with lung cancer for treatment with targeted tyrosine kinase inhibitors: American society of clinical oncology endorsement of the college of American Pathologists/International Association for the Study of Lung Cancer/Association for Molecular Pathology Clinical Practice Guideline Update. J Clin Oncol. (2018) 36 911–9. 10.1200/JCO.2017.76.7293 29401004

[7] Lindeman NI Cagle PT Aisner DL Arcila ME Beasley MB Bernicker EH. Updated molecular testing guideline for the selection of lung cancer patients for treatment with targeted tyrosine kinase inhibitors: guideline from the college of American pathologists, the international association for the study of lung cancer, and the association for molecular pathology. Arch Pathol Lab Med. (2018) 142:321–46. 10.5858/arpa.2017-0388-CP 29355391

[8] Wu J Lu AD Zhang LP Zuo YX Jia YP. [Study of clinical outcome and prognosis in pediatric core binding factor-acute myeloid leukemia]. Chin Med J. (2019) 40:52–7. 10.3760/cma.j.issn.0253-2727.2019.01.010 30704229 PMC7351698

[9] Lega IC Lipscombe LL. Review: diabetes, obesity, and cancer-pathophysiology and clinical implications. Endocr Rev. (2020) 41:bnz014. 10.1210/endrev/bnz014 31722374

[10] Yang J Yang C Shen H Wu W Tian Z Xu Q. Discovery and validation of PZP as a novel serum biomarker for screening lung adenocarcinoma in type2 diabetes mellitus patients. Cancer Cell Int. (2021) 21:162. 10.1186/s12935-021-01861-8 33691685 PMC7945354

[11] Khateeb J Fuchs E Khamaisi M. Diabetes and lung disease: a neglected relationship. Rev Diabet Stud. (2019) 15:1–15. 10.1900/RDS.2019.15.1 30489598 PMC6760893

[12] Raguraman R Srivastava A Munshi A Ramesh R. Therapeutic approaches targeting molecular signaling pathways common to diabetes, lung diseases and cancer. Adv Drug Deliv Rev. (2021) 178:113918. 10.1016/j.addr.2021.113918 34375681 PMC8556346

[13] Harlid S Van Guelpen B Qu C Gylling B Aglago EK Amitay EL. Diabetes mellitus in relation to colorectal tumor molecular subtypes: a pooled analysis of more than 9000 cases. Int J Cancer. (2022) 151:348–60. 10.1002/ijc.34015 35383926 PMC9251811

[14] Xu F Jin J Guo J Xu F Chen J Liu Q. The clinical characteristics, gene mutations and outcomes of myelodysplastic syndromes with diabetes mellitus. J Cancer Res Clin Oncol. (2024) 150:71. 10.1007/s00432-023-05591-4 38305890 PMC10837231

[15] Bermúdez-Hernández GA Pérez-Martínez D Ortiz-León MC Muñiz-Salazar R Licona-Cassani C Zenteno-Cuevas R. Mutational dynamics related to antibiotic resistance in *M. tuberculosis* isolates from serial samples of patients with tuberculosis and type 2 diabetes mellitus. Microorganisms. (2024) 12:324. 10.3390/microorganisms12020324 38399727 PMC10892438

[16] Siegel RL Giaquinto AN Jemal A. Cancer statistics, 2024. CA Cancer J Clin. (2024) 74:12–49. 10.3322/caac.21820 38230766

[17] Wang M Hu RY Wu HB Pan J Gong WW Guo LH. Cancer risk among patients with type 2 diabetes mellitus: a population-based prospective study in China. Sci Rep. (2015) 5:11503. 10.1038/srep11503 26082067 PMC4469976

[18] Morrison M. Pancreatic cancer and diabetes. Adv Exp Med Biol. (2012) 771:229–39. 10.1007/978-1-4614-5441-0_8 23393682

[19] American Cancer Society. Lung Cancer (Non-Small Cell). (2011). Available at: http://www.cancer.org/Cancer/LungCancer-NonSmallCell/DetailedGuide/non-small-cell-lung-cancer-what-is-non-small-celllung-cancer (accessed 15 September 2011).

[20] Bach PB. Smoking as a factor in causing lung cancer. JAMA. (2009) 301:539–41. 10.1001/jama.2009.57 19190320

[21] Lee YJ Cho BC Jee SH Moon JW Kim SK Chang J. Impact of environmental tobacco smoke on the incidence of mutations in epidermal growth factor receptor gene in never-smoker patients with non-small-cell lung cancer. J Clin Oncol. (2010) 28:487–92. 10.1200/JCO.2009.24.5480 20008630

[22] Miller VA Kris MG Shah N Patel J Azzoli C Gomez J. Bronchioloalveolar pathologic subtype and smoking history predict sensitivity to gefitinib in advanced non-small-cell lung cancer. J Clin Oncol. (2004) 22:1103–9. 10.1200/JCO.2004.08.158 15020612

[23] Shigematsu H Lin L Takahashi T Nomura M Suzuki M Wistuba II. Clinical and biological features associated with epidermal growth factor receptor gene mutations in lung cancers. J Natl Cancer Inst. (2005) 97:339–46. 10.1093/jnci/dji055 15741570

[24] Choi JR Park SY Noh OK Koh YW Kang DR. Gene mutation discovery research of non-smoking lung cancer patients due to indoor radon exposure. Ann Occup Environ Med. (2016) 28:13. 10.1186/s40557-016-0095-2 26985396 PMC4793700

[25] Jemal A Miller KD Ma J Siegel RL Fedewa SA Islami F. Higher lung cancer incidence in young women than young men in the United States. N Engl J Med. (2018) 378:1999–2009. 10.1056/NEJMoa1715907 29791813 PMC7717174

[26] Chapman AM Sun KY Ruestow P Cowan DM Madl AK. Lung cancer mutation profile of EGFR, ALK, and KRAS: Meta-analysis and comparison of never and ever smokers. Lung Cancer. 102:122–34. 10.1016/j.lungcan.2016.10.010 27987580

[27] Dang AH Tran VU Tran TT Thi Pham HA Le DT Nguyen L. Actionable mutation profiles of non-small cell lung cancer patients from vietnamese population. Sci Rep. (2020) 10:2707. 10.1038/s41598-020-59744-3 32066856 PMC7026432

[28] Chen YJ Roumeliotis TI Chang YH Chen CT Han CL Lin MH. Proteogenomics of non-smoking lung cancer in East Asia delineates molecular signatures of pathogenesis and progression. Cell. (2020) 182:226–44.e17. 10.1016/j.cell.2020.06.012 32649875

[29] Suda K Tomizawa K Mitsudomi T. Biological and clinical significance of KRAS mutations in lung cancer: an oncogenic driver that contrasts with EGFR mutation. Cancer Metastasis Rev. (2010) 29:49–60. 10.1007/s10555-010-9209-4 20108024

[30] Hua X Hyland PL Huang J Song L Zhu B Caporaso NE. MEGSA: a powerful and flexible framework for analyzing mutual exclusivity of tumor mutations. Am J Hum Genet. (2016) 98:442–55. 10.1016/j.ajhg.2015.12.021 26899600 PMC4800034

[31] D'Angelo SP Pietanza MC Johnson ML Riely GJ Miller VA Sima CS. Incidence of EGFR exon 19 deletions and L858R in tumor specimens from men and cigarette smokers with lung adenocarcinomas. J Clin Oncol. (2011) 29:2066–70. 10.1200/JCO.2010.32.6181 21482987 PMC3296671

[32] Hao F Gu L Zhong D. TP53 mutation mapping in advanced non-small cell lung cancer: a real-world retrospective cohort study. Curr Oncol. (2022) 29:107411–7419. 10.3390/curroncol29100582 36290859 PMC9599964

[33] Soo RA Reungwetwattana T Perroud HA Batra U Kilickap S Tejado Gallegos LF. Prevalence of EGFR mutations in patients with resected stages I to III NSCLC: results from the EARLY-EGFR study. J Thorac Oncol. (2024) 19:1449–59. 10.1016/j.jtho.2024.06.008 38880172

[34] Li S Li X. Analysis of EGFR, KRAS and PIK3CA gene mutation rates and clinical distribution in patients with different types of lung cancer. World J Surg Oncol. (2021) 19:1197. 10.1186/s12957-021-02315-1 34217313 PMC8254946

[35] White V Bergin RJ Thomas RJ Whitfield K Weller D. The pathway to diagnosis and treatment for surgically managed lung cancer patients. Fam Pract. (2020) 37:234–41. 10.1093/fampra/cmz064 31665265

[36] Hamouz M Hammouz RY Bajwa MA Alsayed AW Orzechowska M Bednarek AK. A functional genomics review of non-small-cell lung cancer in never smokers. Int J Mol Sci. (2023) 24:13314. 10.3390/ijms241713314 37686122 PMC10488233

[37] Soria JC Ohe Y Vansteenkiste J Reungwetwattana T Chewaskulyong B Lee KH. Osimertinib in untreated EGFR-mutated advanced non-small-cell lung cancer. N Engl J Med. (2018) 378:113–25. 10.1056/NEJMoa1713137 29151359

[38] Lu S Dong X Jian H Chen J Chen G Sun Y. AENEAS: A randomized phase III trial of aumolertinib versus gefitinib as first-line therapy for locally advanced or metastatic non-small-cell lung cancer with EGFR exon 19 deletion or L858R mutations. J Clin Oncol. (2022) 40:3162–71. 10.1200/JCO.21.02641 35580297 PMC9509093

[39] Abudawood M Tabaasum H Almaarik B Aligohi A. Interrelationship between oxidative stress, DNA damage and cancer risk in diabetes (Type 2) in Riyadh, KSA. Saudi J Biol Sci. (2020) 27:177–83. 10.1016/j.sjbs.2019.06.015 31889833 PMC6933234

[40] Wu D Hu D Chen H Shi G Fetahu IS Wu F. Glucose-regulated phosphorylation of TET2 by AMPK reveals a pathway linking diabetes to cancer. Nature. (2018) 559:637–41. 10.1038/s41586-018-0350-5 30022161 PMC6430198

[41] Kim DS Scherer PE. Obesity, diabetes, and increased cancer progression. Diab Metab J. (2021) 45:799–812. 10.4093/dmj.2021.0077 34847640 PMC8640143

[42] Chen X Kang S Bao Z. Effects of glimepiride combined with recombinant human insulin injection on serum IGF-1, VEGF and TRACP-5b oxidative stress levels in patients with type 2 diabetes mellitus. Evid Based Complement Alternat Med. (2022) 2022:4718087. 10.1155/2022/4718087 35571731 PMC9106459

